# Atrial fibrillation before and after transcatheter aortic valve implantation: an intertwine between survival and quality of life

**DOI:** 10.2459/JCM.0000000000001580

**Published:** 2023-12-08

**Authors:** Crina Ioana Radulescu, Ovidiu Chioncel, Marco Metra, Marianna Adamo

**Affiliations:** a Cardiology, ASST Spedali Civili and Department of Medical and Surgical Specialties, Radiological Sciences, and Public Health, University of Brescia, Brescia, Italy; b University of Medicine ‘Carol Davila’; c Emergency Institute for Cardiovascular Diseases ‘Prof C C Iliescu’, Bucharest, Romania

Aortic stenosis is the most common and prognostically relevant valvular heart disease because of the high prevalence in an increasingly older population and its negative consequences on morbidity and mortality.^1,2^ This leads to a need for intervention, whether surgical or transcatheter^3^ transcatheter aortic valve implantation (TAVI) emerged more than 20 years ago, initially reserved only for extremely old, frail patients with a prohibitive surgical risk. In recent years, randomized trials have provided evidence to sustain the noninferiority of TAVI as compared with surgery, even in intermediate-risk and low-risk patients^4,5^ with new generation devices being associated with a very low rate of complications.^6,7^

Atrial fibrillation is the most common cardiac arrhythmia and a major cause of heart failure hospitalization.^8^ The significant three-fold increase in prevalence over the last 50 years poses important challenges in better preventing atrial fibrillation and its complications.^9^ Atrial fibrillation is present in 15% of patients with severe aortic stenosis and in up to 50% of those undergoing TAVI.^10^ But most importantly, in connection with the mechanisms causing and maintaining atrial fibrillation, the underlying atrial cardiomyopathy is worth mentioning as the common risk factors are also contributing to aortic stenosis progression.^11^

Notably, there are data supporting atrial remodeling as a consequence of the abnormal hemodynamics produced by aortic stenosis independently of the presence of atrial fibrillation. Left atrium volumes are higher in aortic stenosis patients compared with controls and decrease after valvular correction.^12^ Another common cause of atrial cardiomyopathy is chronic heart failure^13^ with a particular and complex phenotype of atrial fibrosis that appears to occur early in the course of heart failure.^14^ As aortic stenosis predominantly manifests as heart failure with preserved ejection fraction, it could be assumed that both valvular heart disease and heart failure contribute to the development of atrial cardiomyopathy. In this context, prevention of atrial fibrillation may also prevent heart failure.^15^

Arrotti *et al.*^16^ conducted a multicentered, observational, retrospective study, which enrolled 759 patients undergoing TAVI between 2012 and 2022 with the aim of evaluating the association between atrial fibrillation and both short-term and long-term outcomes. The median follow-up was 3.2 years. Patients were stratified according to their history of atrial fibrillation: no previous atrial fibrillation (68.2%) and preexisting atrial fibrillation (31.8%). They were equally distributed by gender and comorbidities, and had a mean age of 82.8 years. As for the in-hospital events, at the adjusted logistic regression analysis, patients with history of atrial fibrillation showed significantly higher rates of acute kidney injury (AKI) [adjusted odds ratio (OR) 1.65, 95% confidence interval (CI) 1.13–2.41] and major bleedings (adjusted OR 1.77, 95% CI 0.99–3.16). At discharge, the percentage of patients with atrial fibrillation had increased to 36.6% because of new-onset atrial fibrillation. The occurrence of the composite outcomes (all-cause death/cardiovascular hospitalizations and all-cause death/all-cause hospitalizations) was higher in patients with atrial fibrillation versus those without (35.6 vs. 38.2%). Also, the unadjusted Cox regression analysis showed that atrial fibrillation at discharge was associated with a higher risk of the primary outcome (hazard ratio 1.47, 95% CI 1.13–1.91) and the secondary outcome of all-cause death/all-cause hospitalization (hazard ratio 1.42, 95% CI 1.10–1.83) and hospitalization alone (hazard ratio 1.7, 95% CI 1.23–1.37). No significant association was observed between atrial fibrillation at discharge and cardiovascular events.

The authors need to be congratulated for shedding light on the interplay between atrial fibrillation and aortic stenosis treatment. Indeed, data available so far regarding the impact of atrial fibrillation on mortality after TAVI have reported conflicting results. In a meta-analysis, Sannino *et al.*^17^ found that only preexisting atrial fibrillation was associated with increased mortality, whereas Gargiulo *et al.*^18^ showed that new-onset atrial fibrillation led to an increase in late mortality. Both atrial fibrillation types have been linked to an excess in early mortality.^19,20^ In a recent sub-analysis from the PARTNER 3 trial, preexistent atrial fibrillation in low-risk patients undergoing TAVI was found to be associated with higher rates of death, stroke or rehospitalization, but not all-cause death (3.8 vs 2.6%, *P* = 0.45).^21^ Atrial fibrillation may also impact on quality of life. Almost 40% of patients over 65 years old had impaired quality of life related to atrial fibrillation and the geriatric factors associated were falls, frailty, depression, COPD, rhythm control and anticoagulant therapy.^22^ In this regard, atrial fibrillation may be considered a potential factor preventing quality of life improvement in old patients undergoing TAVI. On the other hand, Laenens *et al.*^23^ reported data from 2849 patients with severe aortic stenosis (mean age 72 years), 24% of them had a history of atrial fibrillation. Median follow-up was 60 months. Even if patients with both aortic stenosis and atrial fibrillation have reduced survival compared with those in sinus rythm, when correcting for indexed left atrial volume, *E*/*e′* or both, atrial fibrillation was no longer associated with all-cause mortality. These results highlight that the negative outcomes may not be associated with atrial fibrillation per se, which especially in younger patients could be a by-stander, but with the underlying structural abnormalities found in aortic stenosis and atrial cardiomyopathy. With the rapidly expanding use of TAVI in young patients, further studies are urgently required to better understand the role of atrial fibrillation in these populations in terms of both hard endpoints and patient-reported outcomes (Fig. 1).

**Fig. 1:**
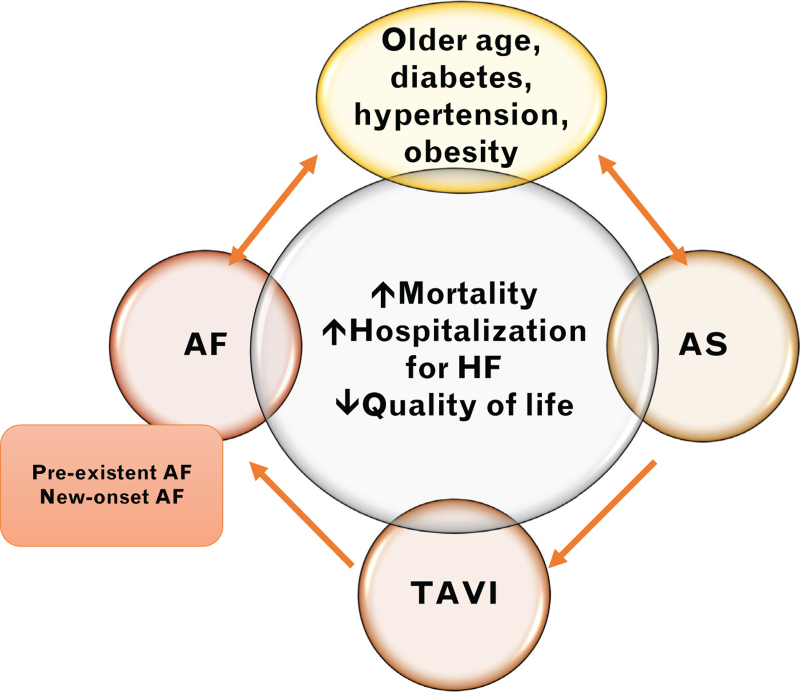
Interplay between cardiovascular risk factors, atrial fibrillation and aortic stenosis.

## Conflicts of interest

There are no conflicts of interest.
